# Plastic Fruit Stickers in Industrial Composting—Surface and Structural Alterations Revealed by Electron Microscopy and Computed Tomography

**DOI:** 10.1021/acs.est.3c08734

**Published:** 2024-04-10

**Authors:** Max Groß, Matthias Mail, Olivia Wrigley, Rafaela Debastiani, Torsten Scherer, Wulf Amelung, Melanie Braun

**Affiliations:** † Institute of Crop Science and Resource Conservation (INRES), Soil Science and Soil Ecology, University of Bonn, Nussallee 13, 53115 Bonn, Germany; ‡ Institute of Nanotechnology (INT), Karlsruhe Institute of Technology (KIT), Kaiserstr. 12, 76131 Karlsruhe, Germany; § Karlsruhe Nano Micro Facility (KNMFi), Karlsruhe Institute of Technology (KIT), Hermann-von-Helmholtz-Platz 1, 76344 Eggenstein-Leopoldshafen, Germany

**Keywords:** micro-CT, nano-CT, deep learning segmentation, price look-up sticker, FTIR, degradation, microplastic

## Abstract

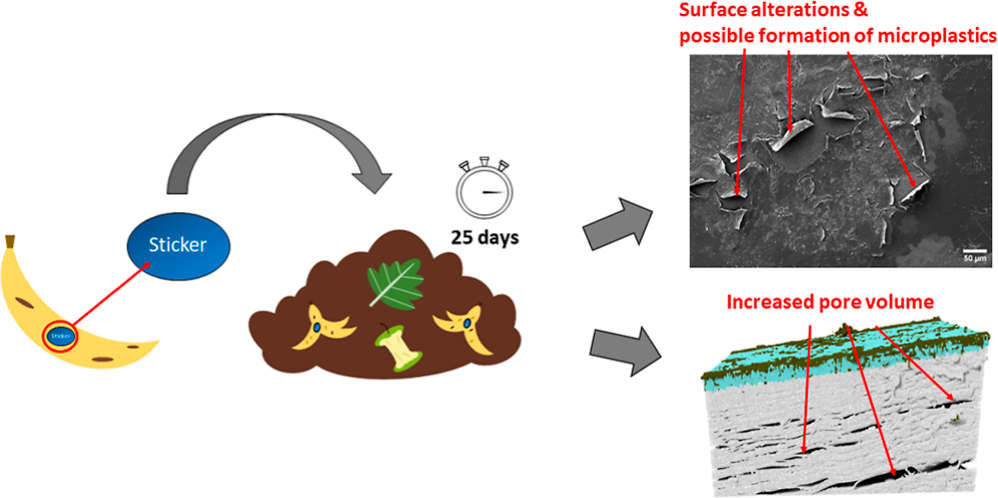

Often large quantities of plastics are found in compost, with price look-up stickers being a major but little-explored component in the contamination path. Stickers glued to fruit or vegetable peels usually remain attached to the organic material despite sorting processes in the composting plant. Here, we investigated the effects of industrial composting on the structural alterations of these stickers. Commercial polypropylene (PP) stickers on banana peels were added to a typical organic material mixture for processing in an industrial composting plant and successfully resampled after a prerotting (11 days) and main rotting step (25 days). Afterward, both composted and original stickers were analyzed for surface and structural changes via scanning electron microscopy, Fourier-transform infrared spectroscopy, and micro- and nano-X-ray computed tomography (CT) combined with deep learning approaches. The composting resulted in substantial surface changes and degradation in the form of microbial colonization, deformation, and occurrence of cracks in all stickers. Their pore volumes increased from 16.7% in the original sticker to 26.3% at the end of the compost process. In a similar way, the carbonyl index of the stickers increased. Micro-CT images additionally revealed structural changes in the form of large adhesions that penetrated the surface of the sticker. These changes were accompanied by delamination after 25 days of composting, thus overall hinting at the degradation of the stickers and the subsequent formation of smaller microplastic pieces.

## Abstract 2

There is no research on how food stickers change during industrial composting. By applying SEM, FTIR, and for the first time, micro- and nano-CT analysis, we were able to detect surface as well as structural changes of fruit stickers made of conventional plastic during composting. The increase in pore volume, crack formation, and detachment of smaller plastic items lead to the conclusion that the degradation of conventional plastic during industrial composting is a source of micro- and submicron plastics.

## Introduction

Compost, the most widely used soil amendment in the world,^1^ contains varying amounts of plastics.^2−4^ A main source of this plastic contamination are price look-up stickers, used internationally for the marketing and labeling of food.^5,6^ These fruit or vegetable stickers, usually made of vinyl or (conventional) plastic, are glued on the peel of various foods and frequently remain on the food material ending up in the organic waste.^7,8^ Due to their small size and thickness, these stickers often pass screening processes in composting plants,^7,9^ although there are currently no estimates of how many stickers end up in composting facilities. Assuming that the average banana weighs 120 g and that one of five bananas receives a sticker, 5.8 million tons of bananas imported in the EU in 2021^10^ would have resulted in about 9.7 × 10^9^ stickers. With an average sticker weight of 0.02 g, this would amount to more than 190 t of plastic per year. These stickers are used not only in the EU but also in other countries around the world,^5^ where they presumably also contribute to the plastic pollution of compost.^11^ In the USA, for example, the US Environmental Protection Agency (EPA) identified these stickers as a major contributor to plastic in compost in its 2021 report.^8^

Composting is a management method used worldwide to treat organic components from solid waste.^12^ The composting process, including the type of the composting system and the composting conditions, as well as the composition of the waste, differs within a country and also on a larger scale between regions of the world:^12−14^ In total, 15% of the municipal solid waste is composted in the EU countries, 8.8% in the USA, <6% in Japan, and <2% in China.^15^ Regardless of the type of composting, conventional plastic and thus most fruit stickers will generally not completely degrade but may undergo surface or structural changes through abiotic, biotic, and mechanical degradation processes.^16^ Such alterations may affect the further fate of plastics when they enter the soil via compost application. Thermo-oxidative and hydrolytic degradation are favored in industrial composting when sufficient oxygen and/or moisture are present,^17^ as temperatures may exceed 80 °C.^12^ Such abiotic degradation processes can increase the surface area of plastic particles^16^ and incorporate hydrophilic groups such as carbonyl, carboxyl, or ester groups, which can thus foster biotic degradation by bacteria, fungi, and biofilms.^18,19^ However, the alteration processes to which such stickers are subjected, i.e., surface and structural changes, have not yet been studied.

Surface imaging techniques via scanning electron microscopy (SEM) and Fourier-transform infrared spectroscopy (FTIR) are well-established methods of analyzing the effects of composting on plastics.^17,20,21^ Such analyses have been performed for various plastics under different pretreatments and composting conditions.^22−24^ Depending on the plastic type and the conditions, composted plastics show surface alterations and adhesion of microbes to the surface, as well as changes in FTIR spectra to a varying degree.^25−28^ Other methods to reveal surface, as well as structural, changes are high-resolution cross-sectional imaging techniques, such as micro-X-ray computed tomography (micro-CT) and nano-X-ray computed tomography (nano-CT). Both methods are established techniques in a variety of scientific fields^29−31^ but are rarely used for plastic analyses. Bittner and Endres^32^ for instance successfully applied micro-CT imaging to reveal surface changes in a polyhydroxybutyrate (PHB) plastic sample covered by biofouling after exposure to a marine environment. Nano-CT, which is capable of achieving voxel resolution in the submicrometric range,^33^ was applied to reveal structural changes of wood–plastic composites after exposure to different environmental conditions.^34−36^ A combination of the methods mentioned above will for the first time allow the detection of surface as well as structural changes of the composted stickers.

We hypothesized that industrial composting leads to surface and structural changes in fruit stickers made from conventional plastic. To elucidate these small-scale structural changes in the fruit stickers, made from conventional polypropylene (PP), we placed them on banana peels and subjected them to industrial tunnel composting. Afterward, we analyzed the original and composted stickers using SEM, FTIR, and CT techniques. We explicitly did not aim to quantify the “degradation” of the stickers in terms of the total amount of plastic, as we already knew from visual inspection and reports from facility owners that composting of conventional plastic does not typically result in significant, measurable weight loss.

## Materials and Methods

### Compost Trial

Industrially produced stickers, made of PP with water-based ink and acrylic-based adhesive, were attached to banana peels to represent a realistic disposal scenario. These were then placed in cylindrical stainless-steel containers with wide openings to allow for water infiltration and exchange with the organic waste in the compost tunnel (Figure S1). To ensure realistic conditions, i.e., mixing with other organic material during composting, the containers were also filled with home-made, plastic-free organic waste. The self-generated waste was based on the typical composition of organic waste in the tunnel and consisted of green cuttings and typical organic household waste items (fruit and vegetable pieces/peelings) and then thoroughly mixed until a homogeneous material was formed. Subsequently, the containers were closed with galvanized mesh, wires, and cable ties and attached to an 8 m galvanized chain. Galvanized metal was used to avoid rust and breakage during composting. A total of 12 containers were prepared, each containing four stickers, to ensure that there were sufficient replicates after composting, taking into account possible losses and damage during the process. The subsequent composting trial was conducted at a nearby industrial composting facility that processes organic household waste in tunnel composting. These tunnels (25 m length, 5.7 m width, and 5 m height) were filled with organic waste to a height of 3 m, corresponding to an input quantity of 286 t of organic waste per tunnel. The 12 containers were placed within the organic waste in the tunnel (Figure S2). The composting process was divided into a prerotting (11 days) and a main rotting (14 days) phase, accounting for a total of 25 days of composting. During composting, irrigation, and air circulation of the organic material were ensured via built-in systems. After prerotting, the now partially rotted material was transferred to a decompactor and loaded into a second tunnel for the subsequent main rotting process. The temperature profile (Figure S3) showed a peak of 60 °C during prerotting and 70 °C at the beginning of main rotting; thereafter, the temperature decreased steadily until it reached 20 °C (Figure S3).

### Sampling

The first sampling took place at the end of prerotting (11 days of composting). During the unloading of the tunnel, all containers were removed, and the first four containers were sampled. The remaining eight containers were transferred to the second tunnel along with the prerotted material for the main rotting phase (Figure S4). At the end of the main rotting phase (total of 25 days of composting), the remaining set of containers was sampled. All stickers were then stored in a refrigerator at 0–5 °C until further processing.

### Preparation for SEM and CT Analysis

To ensure the preservation of the microbial structure and avoid further alterations of the stickers, they were fixed using a phosphate-buffered saline (PBS) buffer and a fixative solution (PBS buffer and glutaraldehyde; detailed description in the Supporting Information).

For SEM analyses, a section (approximately 0.5 cm^2^) of each sticker was isolated and placed on conventional SEM pin stubs (Plano GmbH, Wetzlar, Germany) using conductive silver paint (Plano GmbH, Wetzlar, Germany) for mounting. The surfaces of the stickers were then coated with a 7 nm-thick platinum layer using a sputter coater (Cressington Coating System 328, Cressington Coating Systems, Watford, England). In addition to the 12 composted stickers (one from each container), four original non-composted stickers were also prepared, which were previously stored in the dark at room temperature.

For micro-CT analyses, sections of the original and 25 day composted stickers were isolated and placed on specific sample holders. The original stickers were prepared with the release paper still attached for ease of analysis. For nano-CT analyses, samples of the original and 11 day composted stickers were prepared with a scalpel and samples of the 25 day composted sticker were cut with a laser (microPREP PRO, 3D-Micromac AG, Chemnitz, Germany) in slices of approximately 60 μm thickness and then placed on the respective sample holders (Table S1).

### Data Collection

Overview SEM images were acquired for one sticker replicate of each container (via a ZEISS Leo-1530 and ZEISS Auriga 60, Carl Zeiss AG, Oberkochen, Germany). For 7 of the 12 composted stickers, two randomly selected areas were imaged (via ZEISS Leo-1530) with varying pixel sizes from 2867 to 19.11 nm, resulting in imaged areas of 5.818 × 10^7^ μm^2^ (58.18 mm^2^) for the largest pixel size and 2496.73 μm^2^ for the smallest pixel size, respectively. For the remaining five composted stickers and the four original stickers, an area of 0.94 mm^2^, with a pixel size of 19.1 nm, was imaged using a ZEISS Auriga 60. For more detailed imaging of a 25 day composted sticker, an environmental scanning electron microscope (Phillips XL30 ESEM-FEG) was used. All imaging was carried out using an acceleration voltage of 5 kV and BSE and SE detectors.

Micro-CT imaging was performed with a working voltage of 50 kV and power of 4 W, using a ZEISS Xradia 520 Versa. The samples were rotated 360°, and 2001 projections were acquired with an acquisition time of 10 s. Nano-CT scans were performed using the ZEISS Xradia 810 Ultra X-ray microscope. This system uses a semimonochromatic X-ray beam from a chromium anode source (energy of 5.4 keV) and a sequence of optics to achieve a pixel size of 64 nm within a field of view of 65 μm. The samples were scanned over 180° with an acquisition time ranging from 20 to 50 s, acquiring 501 to 901 projections, in Zernike phase contrast mode. The specific parameters for each sample are described in Table S1.

FTIR spectra of the stickers were recorded in the range of 4000–600 cm^–1^ with a resolution of 4 cm^–1^ and 128 scans using the Bruker LUMOS II FTIR microscope (Bruker Corporation, Billerica, United States) in attenuated total reflection (ATR) mode.

### Data Analysis

Image analysis and data processing of the SEM images were carried out using the software Fiji 2.9.0.^37^ FTIR spectra were evaluated in OPUS version 8.7.41 (Bruker Corporation, Billerica, Unites States) and the carbonyl index (CI) was calculated based on the FTIR spectra according to Almond et al.^38^ Briefly, the area under the carbonyl signal (1850–1650 cm^–1^) was divided by the area under the methylene scissoring signal (1500–1420 cm^–1^)^38^ (eq S1).

The arithmetic mean, standard deviation, and boxplots of the carbonyl indices of the three groups were determined. In addition, a Shapiro–Wilk test was performed. As the data were not normally distributed (Shapiro–Wilk test: *p* < 0.05), a Kruskal–Wallis test followed by a posthoc test (Dunn’s test) was then carried out. All statistical analyses were performed in SigmaPlot (Systat Software Inc., San Jose, California, USA). Micro- and nano-CT data sets were reconstructed using the proprietary software Zeiss Scout-and-Scan Reconstructor, a software based on a filtered back projection algorithm, and analyzed using ORS Dragonfly 2022.2.^39^ Data segmentation was performed in Dragonfly’s segmentation wizard by applying the UNet++ model, a neural network model originally developed for medical image segmentation.^40^ The training of the models was repeated until satisfactory results were achieved. For this purpose, the dice similarity coefficient (DSC), the most commonly used metric to assess the validation and performance of the models, was calculated.^41^ The DSC calculates the similarity or overlap between two samples. Its range of values is between 0 and 1, with a value closer to 1 indicating a better segmentation effect.^42^ The DSCs of the UNet++ models (micro-CT) were 0.949 for the original sticker and 0.988 for the 25 day composted sticker. The UNet++ models of the nano-CT data had a DSC of 0.927 for the original sticker, 0.932 for the 11 day composted sticker, and 0.935 for the 25 day composted sticker. In addition, the models were visually checked for agreement with the original CT images. The models were then applied to the respective dataset. The three nano-CT data sets were segmented into five classes: upper part of the sticker, lower part of the sticker, pores, attachments (entirety of material on the surface of the stickers), and background. The micro-CT datasets were segmented into sticker, attachments (entirety of material on the surface of the stickers), background, and additionally for the data set of the original sticker, release paper. Subsequently, volume and surface area (according to Lindblad^43^) of each class were determined by connected component analysis in ORS Dragonfly. Since the sticker samples (nano-CT) were of different sizes, the percentage of the pore volume in the total sticker volume was determined for comparison (eq S2). To rule out edge effects possibly caused by the preparation, the calculations were performed in the center of the stickers as the laser preparation of the sticker, and the resulting heat created large pores in the outer area of the sticker. Stickers prepared with a scalpel did not show these effects. Finally, the segmentation results were displayed as images.

## Results and Discussion

**Figure 1:**
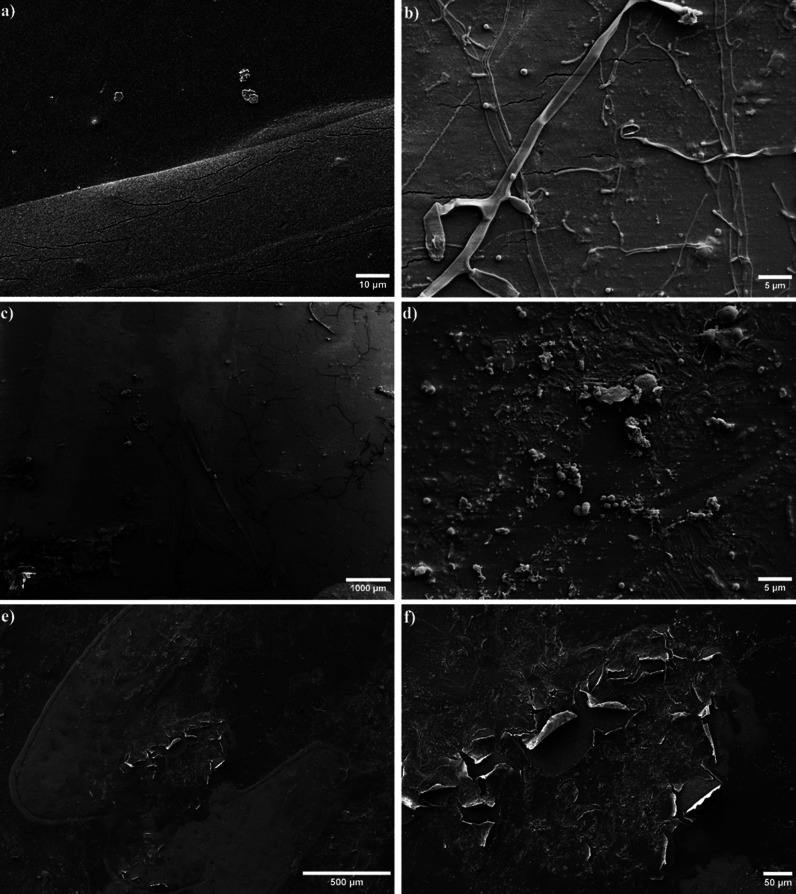
(a) Original, noncomposted sticker with cracks on the print layer and attachments as well as typical dents on the rest of the sticker. (b) Typical small cracks and hyphae on a 25 day composted sticker. (c) Longer, rectangular branched cracks and larger attachments on an 11 day composted sticker. (d) Individual prokaryotic cells and microcolonies on an 11 day composted sticker. (e) General view of delamination on the 25 day composted sticker. Two areas can be seen where delamination has occurred, concentrated on the print. (f) Higher magnification of the larger delaminated area. The delaminated layer appears to be rougher than the underlying layer.

The SEM images of the original, noncomposted stickers showed a variety of particles adhered to the surfaces (Figure S5). However, biological colonization, such as hyphae or conidia, was absent (Figure 1a). The printed areas of the sticker represented an elevation of the surface and appeared to be rougher than the unprinted areas. Within the printed areas, the distribution of the ink resulted in some areas of thin ink coverage next to patches of unprinted areas. All of the original stickers had cracks, but these occurred only in the printed areas and exhibited a maximum length of 20 μm; only one sticker had cracks up to 90 μm long (Figure 1a). The unprinted areas showed only minor surface irregularities, mostly in the form of narrow grooves or minor dents. Some of the surface irregularities on the original stickers may have occurred during production or when the sticker was peeled from the release paper.

In contrast to the original stickers, all of the composted stickers exhibited a variety of surface changes. These surface alterations were not concentrated in any particular area of the stickers and were visible after 11 days of composting (Figure S5). Similarly, various types of attachments such as hyphae, prokaryotic cells, and organic residues of the composted material were found (Figures 1b,d and S5). Differences in the amount or type of surface changes (i.e., cracks, grooves, and dents) between stickers of prerotting and main rotting were, however, not detected. Nevertheless, different types of cracks were visible, which, according to Deng et al.,^44^ can be divided into four main types: line, curve, net, and unclassified, each with further subtypes. The most common subtype found on stickers was short lines (main type line) with sizes ranging from 1 to 3.5 μm (Figure 1b). In addition, switches (subtype of the main type line) were seen as longer cracks, usually branching at right angles and partially interconnected (Figure 1c). Curved lines were also observed; these were often longer and did not have as many branches as switches. Also, unclassified cracks occurred as a mixture of straight and short lines that had neither regular spacing nor any preferred direction.^44^ In addition to cracks, holes (typical <0.5 μm in diameter), scratches, dents, and other irregularities were common. Overall, the composted stickers showed substantial physical surface changes compared to the surfaces of the original stickers. Consequently, we conclude that industrial composting leads to at least a physical surface alteration of fruit stickers made of conventional PP.

To date, there have been no further studies on the effect of composting on fruit stickers. Therefore, comparisons can only be made with studies in which composted PP, the main component of the stickers, was analyzed. PP films showed cracks but also cavities on the surface after 7 months of composting at the laboratory scale (temperatures of 25–43 °C).^45^ Here, pre-treatments by UV and γ radiation prior to composting resulted in significantly more pronounced surface erosion after composting. The macrocracks on the surface after pretreatment served as a starting point for biodegradation, resulting in a 3-fold (UV-radiation) and 6-fold higher (γ-radiation) biodegradation rate compared to the untreated sample after 4 months of composting.^45^ Accordingly, we assume that pre-damaged stickers, e.g., by UV radiation or shredding mechanisms within the compost plant, will experience more surface changes than intact stickers during the composting process. Sholokhova et al.^46^ studied fresh PP food containers as well as PP film packaging after 7 months of windrow composting (temperatures of 30–65 °C). Their SEM analyses revealed surface changes, which were dependending on the thickness of the plastics. The thicker and stiffer PP food containers showed mainly cracks, scratches, and plowing, while the PP film had mainly cracks and holes. These holes, which were mostly <10 μm in diameter, indicate microbial attack during composting^46^ and were also observed on most of the fruit stickers in the present study, although they were smaller (<0.5 μm in diameter), which may also be due to different microscope settings in our study.

In addition to organic residues, microbial colonization, as a starting point for microbial attack, was observed on all composted fruit stickers (Figure S5). Fungal colonization of the sticker surface was already evident after prerotting (11 days); here numerous conidia in connection with hyphae could be found (Figure 1b). The conidia and hyphae varied in size, shape, and structure and some conidia were clumped together (Figure 1b); this was also observed on microplastic particles collected from landfill soils in Kenya.^47^ It is presumed that the fungi produced mucilage that supported adhesion to the plastic surface (Figure S5c).^47^ Individual prokaryotic cells and microorganisms were also present (Figure 1d). In principle, PP is not susceptible to microbial attack, mainly due to its high molecular weight, hydrophobic backbones, high packing density, and the possible addition of antioxidants or stabilizers. However, after abiotic degradation processes, especially oxidation and photodegradation, its hydrophobicity decreases and carbonyl and hydroxyl groups are formed, allowing microorganisms to attach to the surface and subsequently grow using the host polymer as a carbon source, thus leading to further erosion of the polymer surface.^19,20,48^ This microbial community on the surface of plastic particles, also reffered to as plastisphere,^47,49^ may differ from the surrounding compost, indicating species enrichment and selection on plastic particles.^50^ The composition of such plastisphere organisms appears to be plastic-type-specific as the community associated with polyurethane (PU) differed from that of the surrounding compost,^50^ whereas no difference was found between low-density polyethylene (PE) and the bulk compost.^51^ Detailed studies on the microbial colonization during composting have not yet been conducted for fruit stickers or even PP. Therefore, future research focusing on the microbial community on the sticker surfaces and their potential for biodegradation is needed.

The most pronounced surface change observed during this study was the delamination of the printed layer on one sticker after 25 days of composting (Figures 1e,f, and S6). In two areas, approximately 0.3 and 0.1 mm^2^ in size, many plastic fragments in the sizes of 30 and 95 μm partially detached from the bottom layer. These fragments appeared to be rougher than the bottom layer and rolled up to a height of over 27 μm (Figure S6). As the delamination only occured in the area with printing, the cracks that were already present on the original stickers likely served as an initiator.^52^ It is also possible that the printed areas behaved differently from the unprinted areas during composting (e.g., differences in surface roughness or heatability). Although the formation of microplastics and submicrometer plastics by the observed delamination cannot be unequivocally confirmed by SEM, our results suggest that smaller pieces of plastic can be formed and may be released during composting.

The formation and release of smaller particles from conventional plastics during composting have been confirmed in the past. Gui et al.^27^ found that the microplastic content increased during composting, i.e., that the raw material (rural household waste) contained significantly (*P* ≤ 0.05) fewer microplastics in the size of <0.5 mm than the finished compost. To support these results, Gui et al.^27^ conducted a laboratory-scale composting experiment over 30 days for expanded polystyrene (EPS), PE, and PP. The authors found a release of microplastics for all tested materials ranging from 5 to 53 particles on average per piece of plastic. Even more microplastics were released from several types of conventional plastics (HDPE, LDPE, PP, and PS) during industrial windrow composting; here in total, 56–122 released particles per piece of plastic were found.^46^ Both studies revealed the release of microplastics being plastic-type-specific, depending on the properties but also the thickness of the material. In detail, while PP released significantly less microplastic than EPS,^27^ thin PP films released significantly more items than thicker, more rigid PP.^46^ Since the fruit stickers have film-like properties, the observed release of microplastics seems plausible. Remarkably, small plastic particles >50 μm were detached regardless of the thickness of the PP film,^21,40^ and in the laboratory-scale composting, 74% of all particles were in the lower size range of 50 to 500 μm.^27^ For both studies, the lowest detection limit was 50 μm. Since some of the partially detached particles on the sticker surface were smaller than 50 μm (between 30 and 95 μm), we can assume that composting releases an even larger number of particles from plastics that have not yet been analyzed. This may be particulary the case for submicron plastics. In principle, however, the release of plastics during composting and the formation of smaller plastic particles from fruit stickers can also occur due to mechanical decomposition, e.g., during compost pre-treatment or overturning.^21,47^

**Figure 2:**
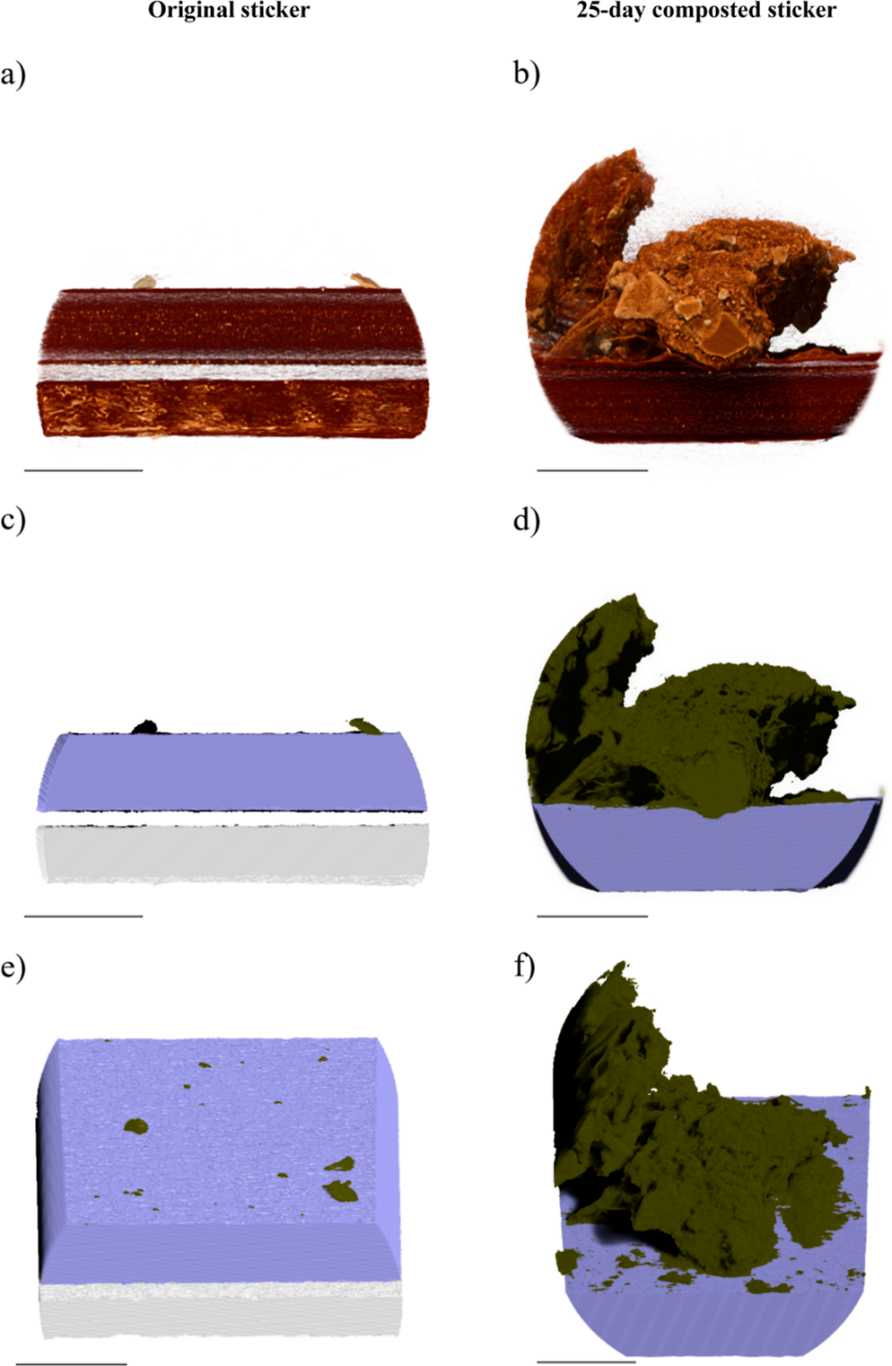
Orthographic 3D projections of the data obtained by micro-CT (scale bar: 100 μm). The left side shows the images of the original sticker. The top image (a) shows a cross section, with the individual layers clearly visible. The images of the segmented data (c,e) show the release paper (white) and the sticker (purple) with only a few particles adhering to its surface (dark green). On the images of the composted sticker (right side, b,d,f), a clear formation of attachments on the surface of the sticker can be seen. These attachments penetrate the sticker and show different X-ray absorption values. Here, too, differentiation of the attachments (dark green) from the sticker (purple) could be achieved with the help of the deep learning approach.

For structural analyses of the stickers, data from micro- and nano-CT were evaluated. Micro-CT analysis of the original sticker showed two structurally distinct layers, namely, the sticker (upper layer) and the release paper (lower layer), which were separated by the adhesive (Figure 2a). After 25 days of composting, the volume of the attachments on the sticker surface was more than 550 times larger (3.52 × 10^6^ μm^3^) than that of the original sticker (6.24 × 10^3^ μm^3^), which, as observed in the SEM images, indicates a significant accumulation of organic material (Figure 2, Table S2). The surface area of the attachments also increased from 4.83 × 10^3^ μm^2^ on the original sticker to 6.49 × 10^5^ μm^2^ on the composted sticker (Table S2). The few particles that adhered to the original sticker neither penetrated the surface nor changed the structure of the sticker (Figure 2a,c). In contrast, the attachments on the composted sticker consisted of different layers and particles, all differing in shape, structure, size, and X-ray absorption (Figure 2b). Based on SEM analysis, we assume that the attachments are composed of microorganisms (e.g., fungi or prokaryotic cells) and residues of the composted material.

Similar mechanisms of microplastic degradation have been demonstrated by micro-CT analyses of a biodegradable PHB sample exposed to a marine environment for several months^32^ and an unspecified microplastic particle collected in the North Atlantic.^53^ Both samples were completely covered with a biofilm consisting of a variety of microorganisms. The cross-sectional image of our 25 day composted sticker showed that some particles had penetrated the surface, causing a change in the structure of the sticker near the surface (Figure 2b,d). This could be due to either biological activity on the surface or mechanical degradation, due to the pressure exerted by the organic material on the sticker.^54^ However, no differentiation can be made between the two processes using micro-CT. Deeper inside the sticker, the structure still appeared to be homogeneous and did not show any changes, breakups, or cracks. In contrast, such cracks on the surface, which usually spread locally and can also increasingly affect the structure of the polymer,^52^ could be detected in the CT data of the PHB samples exposed to the sea. These cracks extended to a depth of about 600 μm. In addition, an increase in surface roughness, due to disintegration events, was observed compared to freshly manufactured PHB.^32^ Similar cracks, several hundred micrometers deep, were also observed on the unidentified microplastic particle, which was exposed to seawater. However, the existing biofilm did not seem to penetrate or change the plastic surface.^53^

**Figure 3:**
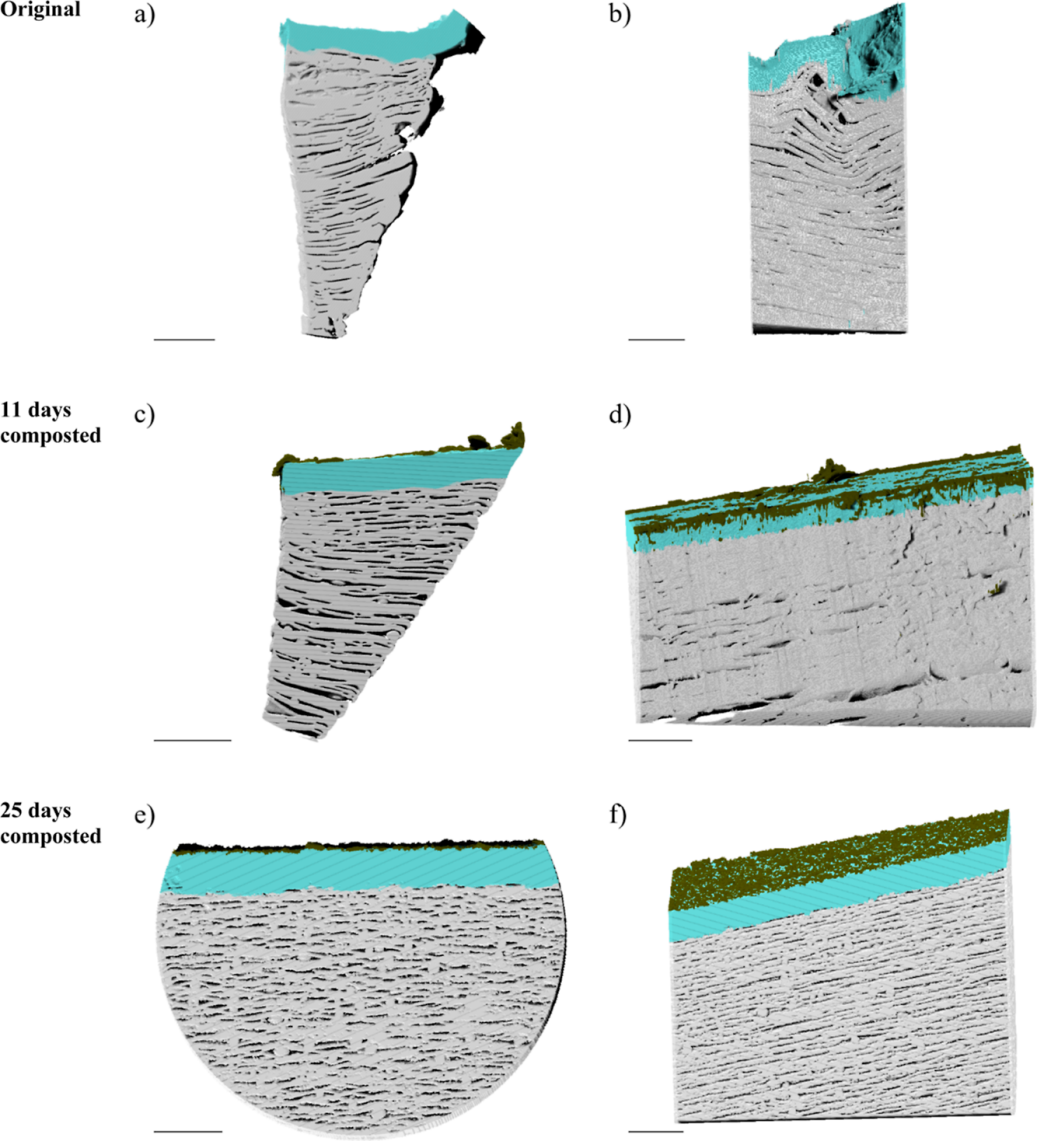
Orthographic 3D projections of the data obtained by nano-CT (scale bar: 10 μm). Cross section of the segmentation results of the original (a,b), the 11 day composted (c,d), and the 25 day composted (e,f) sticker. Dark green, turquoise, and white colors refer to the attachments, the upper part, and the lower part of the sticker, respectively.

Due to the higher resolution by a smaller voxel size of the nano-CT (size: <130 nm/voxel) compared to micro-CT (size: <750 nm/voxel), a more detailed imaging and subsequent differentiation of the stickers into 5 classes could be performed (Figure 3). As already confirmed by the SEM images, the two composted stickers had attachments that were not visible on the original sticker. The proportion of attachments in the total volume was similar at both stages: 4.3% after 11 days and 4.2% after 25 days, respectively. The location on the stickers where the attachments were found varied. While these were mainly concentrated at the edges of the sticker after prerotting, after main rotting, the sticker showed regular attachments evenly distributed over the entire surface (Figure 3). Whether these differences in location are caused by the propagation of different microorganisms cannot be determined from the CT analysis. Due to similar elemental composition, the size of the microorganisms, the smaller field of view scanned, and the scanning resolution for CT analysis, unlike SEM analysis (Figure 1), do not allow distinguishing between microorganisms and other organic residues on the surface of the stickers. It is important to note that the microorganisms are not homogeneously distributed over the stickers (Figure 1d) and that the field of view of the nano-CT corresponds to a small region of 65 μm. However, to be able to further differentiate the attachments on plastic particles in CT images in the future, we would suggest two possible strategies: select the region of interest with high concentration of microorganisms in the SEM image and crop a cylinder from it using focused ion beam (FIB) or carry out several scans of different areas of the sticker in the nano-CT within the field of view of 65 μm to help to select the region with high concentration of attachments. Once the region of interest is selected, a high-resolution nano-CT scan is performed, reaching voxel sizes of 16 nm, but within an even more reduced field of view.

Another major difference between micro-CT and nano-CT images was the visibility of the pores in the bulk of the sticker, the structure of which showed significant differences before and after composting. The original as well as the 11 day composted sticker (Figure 3a–d) had clearly visible large pores, whereas the 25 day composted sticker had smaller but more frequent pores (Figure 3e,f). The upper parts of both the original and the 11 day composted sticker did not show any pores. Exept for the pores at the edge in the upper area of the 25 day composted sticker, which could be attributed to the preparation method, this sticker also showed no pores in the upper area. The percentage of pores by volume within the sticker increased during composting from 16.7% in the original sticker to 21.3% in the 11 day composted sticker and again to 26.3% in the 25 day composted sticker (Table S3). The final pore volume derived from the nano-CT images was up to 10 times higher than those in the other studies using CT imaging. ter Halle et al.,^53^ for instance, found a total crack/pore volume of 3% in the microplastic particle, and even lower pore volumes were observed by Bhagat et al.^55^ These authors did not find any significant difference in pore volume between an untreated (2.4 ± 0.6%) and artificially aged HDPE microplastic sample (1.5 ± 0.2%).

The differences reported for crack development and changes in the pore volume of the different plastic particles are probably due to differences in the stability or structure of the different polymers, especially when considering biodegradable plastics such as PHB. In addition, differences in environmental conditions may also play a role. In particular, the temperature and the degree of photodegradation, the latter being the most important process for the degradation of plastics,^54^ differ between marine environments and composting. During composting, the exposure to UV radiation and thus photochemical degradation is largely hindered, rendering thermo-oxidative degradation as the most common degradation pathway.^17^ In contrast to photodegradation, thermal reactions are not restricted to the plastic surface but affect the bulk polymer.^56^ Weight losses of PP, for example, have been shown to increase from 13 to 18% when composting is prolonged from 4 to 7 months with composting temperatures of up to 43 °C.^45^ An additional weight loss of 5% took place when temperatures reached 70 °C.^57^ In our study, the industrial composting time was substantially shortened, but temperatures in such industrial plants usually exceed 70 °C.^12^ As conventional plastic is not designed to degrade, as required for biodegradable plastics in laboratory tests (e.g., ISO 16929:2021 or ASTM D 640053^58^), the incomplete degradation of plastics and their associated materials in compost is likely to continue.

Another reason for the high pore volumes reported in our study compared to other studies could be the more sensitive technical settings of the CT imaging. The voxel sizes of the earlier studies were 5.61,^32^ 3,^55^ and 1.7 μm/voxel,^53^ i.e., significantly larger than the voxel sizes used here for our nano-CT (<0.13 μm/voxel) and micro-CT (<0.75 μm/voxel) images of the stickers. The finer voxel sizes allow more detailed structures to be seen, although the imaging of smaller areas includes the risk of large cracks with a length of up to 600 μm^32^ being overlooked. To confirm the effects of composting on the fruit stickers, FTIR spectra of the stickers were acquired in addition to SEM and CT analyses. In particular, the CI is a accepted parameter for measuring the changes in physicochemical properties due to the formation of carbonyl species in the range of 1850–1650 cm^–1^ during photo- or thermo-oxidation processes.^38^ Indeed, our samples also showed an increase in the mean CI with increasing composting time from 1.15 ± 0.12 to 1.25 ± 0.24 to 1.45 ± 0.24 (for the original, 11 and 25 day composted stickers, respectively) (Figures S7 and Table S4). Significant differences (*p* < 0.05) between the CI of the three groups could only be determined between the original and the 25 day composted stickers using the Kruskal–Wallis test followed by Dunn’s test. Furthermore, a stretching of the hydroxyl region (3500–3100 cm^–1^) and an increase in the absorbance units at wavenumbers 2951, 2919, 2867, and 2839 were observed with increasing composting time (Figures S8 and S9). This indicates that chain scission, cross-linking, and the formation of new functional groups occurred during the composting process, accompanied by an increase in the C–H bond intensity. Such changes are likely to be caused not only by thermo-oxidation but also by microbial consumption of low molecular weight compounds from the polymer backbone chain.^45,46,59^ Other studies have suggested that during composting, there may be an additional decrease in intrinsic viscosity and weight loss of PP,^45,46,48,60^ which was not monitored here. Fruit stickers are a special case of plastic particles, as they are in direct contact with food and are therefore subject to special guidelines. At the European Union level, the general requirements for food contact materials are laid down in framework regulation EC 1935/2004. Specifically, for plastic materials in direct contact with food, regulation (EU) no. 10/2011 and its latest amendment, regulation (EU) no. 2020/1245, set migration limits for substances that are authorized to be in contact with food contact materials.^61^ Yet, these regulations do not apply to the other materials in fruit stickers, such as adhesives, printing inks, and coatings.^56^ As different amounts and types of adhesives and inks could affect the surface properties of the stickers or serve as a carbon or energy source for microorganisms, fruit sticker degradation can likely not be predicted from laboratory tests using the plastics alone. Future studies should thus not be restricted to certain standard plastic materials but continue to use real environmental plastic mixtures under various complex but also realistic settings. Even different plastic particles of the same type might be found in the environment, differing in their configuration (isotactic, syndiotactic, or atactic) or the manufacturing processes used (extrusion or injection molding as the most common processes), which in turn has a significant influence on the properties of the resulting plastic parts.^62−64^ Elucidating how possible subsequent variations in, for example, the original pore structures of plastic products affect the fragmentation dynamics and structural changes during composting might thus warrant further attention.

## Supporting Information Available

Chain with attached containers; position of containers at the start of prerotting; temperature during composting; position of containers at the start of main rotting; washing and fixing of stickers; overview SEM images of noncomposted and composted stickers; ESEM image of the 25 day composted sticker; boxplots of CI; FTIR spectra of noncomposted and composted stickers; parameters of micro- and nano-CT experiments and data sets; results of connected component analysis of micro-CT data; results of connected component analysis of nano-CT data; values for calculation of CI; calculation of the CI; and calculation of pore volume (PDF)
