# Supplementary material for: Plastic Fruit Stickers in Industrial Composting—Surface and Structural Alterations Revealed by Electron Microscopy and Computed Tomography
Source: Environ Sci Technol. 2024 Apr 10;58(16):7124–32. doi: 10.1021/acs.est.3c08734 (PMC11044595; doi:10.1021/acs.est.3c08734)
Supplement: Supplementary file 1 — es3c08734_si_001.pdf [file es3c08734_si_001.pdf]

Supporting Information for

Plastic fruit stickers in industrial composting – surface alterations revealed by electron microscopy and computed tomography

Max Groß[1*], Matthias Mail[2,3], Olivia Wrigley[1], Rafaela Debastiani[2,3], Torsten Scherer[2,3], Wulf Amelung[1], Melanie Braun[1]

[1]Institute of Crop Science and Resource Conservation (INRES), Soil Science and Soil Ecology, University of Bonn, Nussallee 13, 53115 Bonn, Germany.

[2]Institute of Nanotechnology (INT), Karlsruhe Institute of Technology (KIT), Kaiserstr. 12, 76131 Karlsruhe, Germany.

[3]Karlsruhe Nano Micro Facility (KNMFi), Karlsruhe Institute of Technology (KIT), Hermann-von-Helmholtz-Platz 1, 76344 Eggenstein-Leopoldshafen, Germany.

*Corresponding author: max.gross@uni-bonn.de

Number of pages: 16

Number of figures: 9

Number of texts: 1

Number of tables: 4

Number of equations: 2

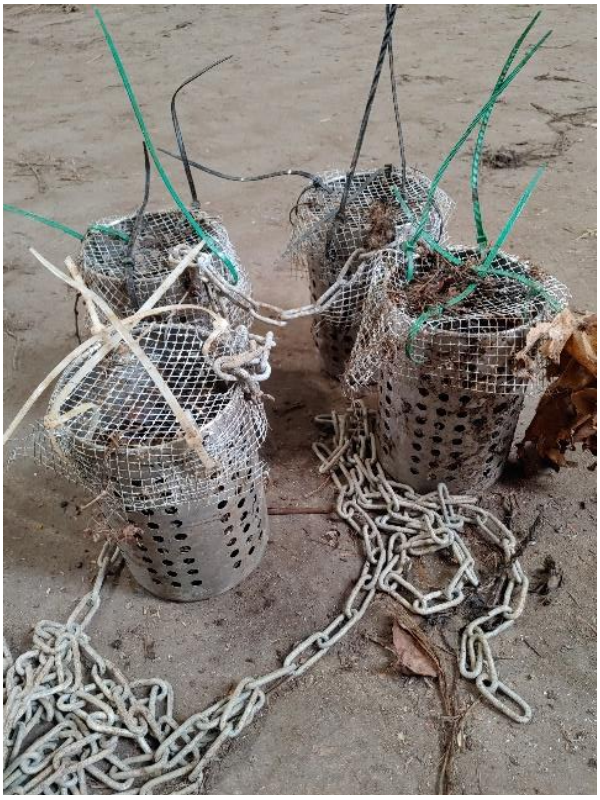

Supplementary Figure S1: Chain with the attached containers after the first rotting process (11 days of composting).

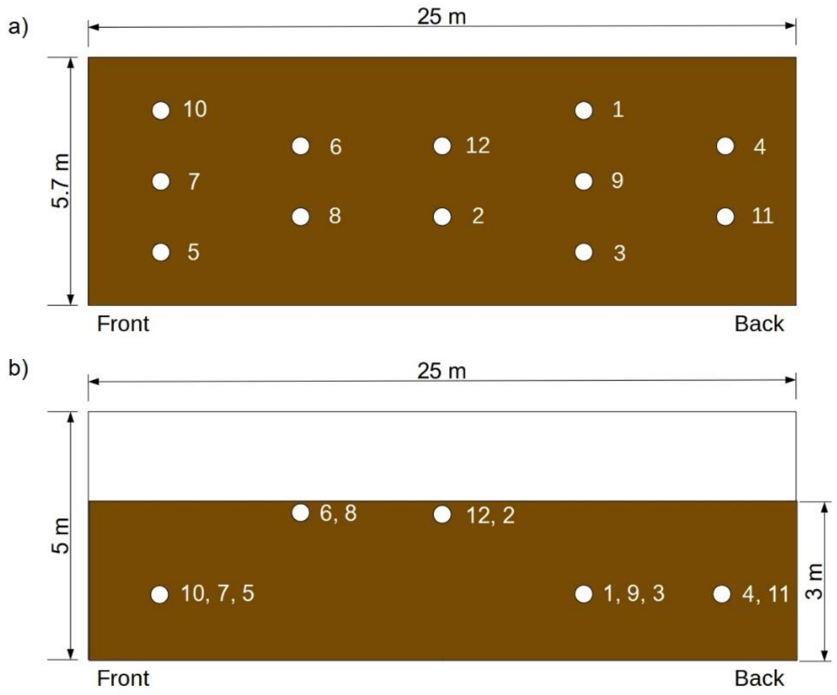

Supplementary Figure S2: a) Top and b) side view on the position of the containers inside the compost tunnel at the start of the pre-rotting process.

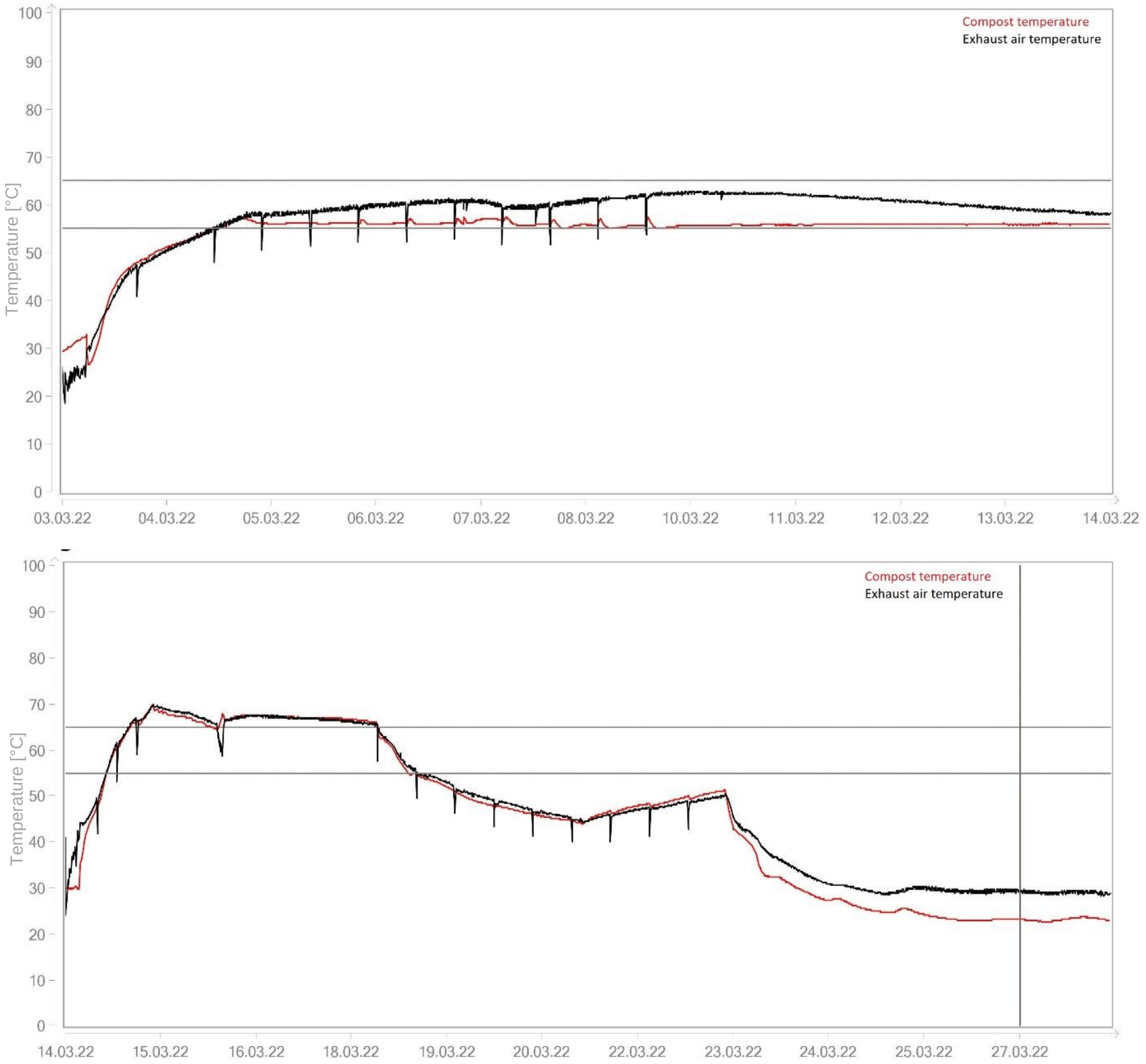

Supplementary Figure S3: Temperature course of the compost and exhaust air during pre-rotting (top) and main rotting (bottom).

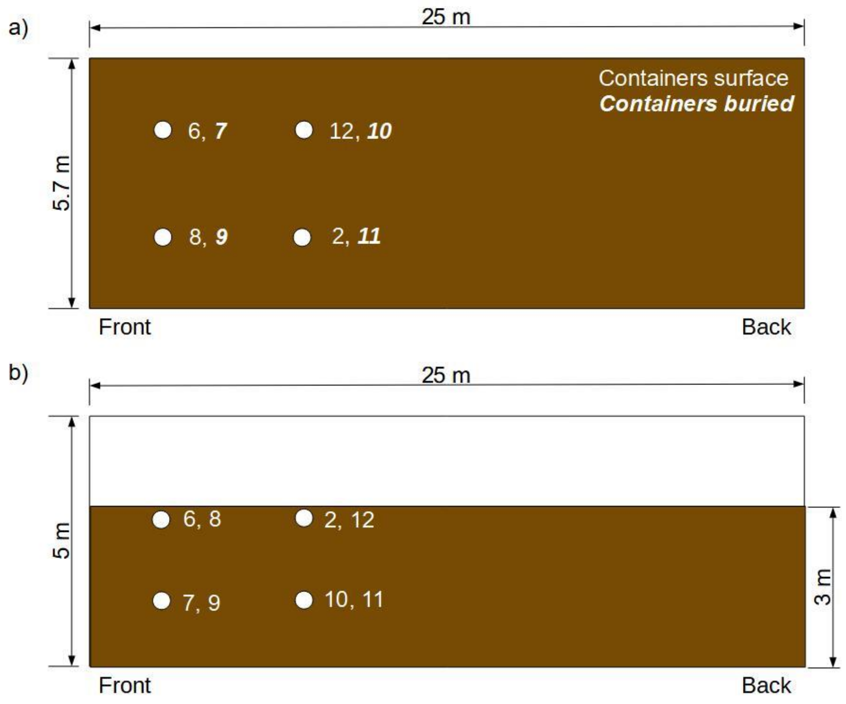

Supplementary Figure S4: a) Top and b) side view on the position of the containers inside the compost tunnel at the start of the main rotting process.

Washing and fixing of the stickers

First, the stickers were washed with PBS buffer, consisting of 8.0 g sodium chloride (Merck KGaA, Darmstadt, Germany), 0.2 g potassium chloride (Sigma-Aldrich Laborchemikalien GmbH, Seelze, Germany), 1.42 g disodium hydrogen phosphate (Merck KGaA, Darmstadt, Germany), and 0.27 g potassium dihydrogen phosphate (Merck KGaA, Darmstadt, Germany) diluted in 1 L distilled water. The stickers were then transferred to a fixing solution (pH 7.4) consisting of PBS buffer and 2.5 % glutaraldehyde. After an incubation period of 60 minutes, the stickers were again placed in PBS buffer for 20 minutes. Then the stickers were transferred to 70 % ethanol (VWR international, Radnor, USA) for 60 minutes, and to 100 % ethanol (VWR international, Radnor, USA) for another 60 minutes. The final step was the addition of hexamethyldisilazane (Merck KGaA, Darmstadt, Germany). The stickers were left under the fume hood until all the hexamethyldisilazane had evaporated and then stored in a desiccator.

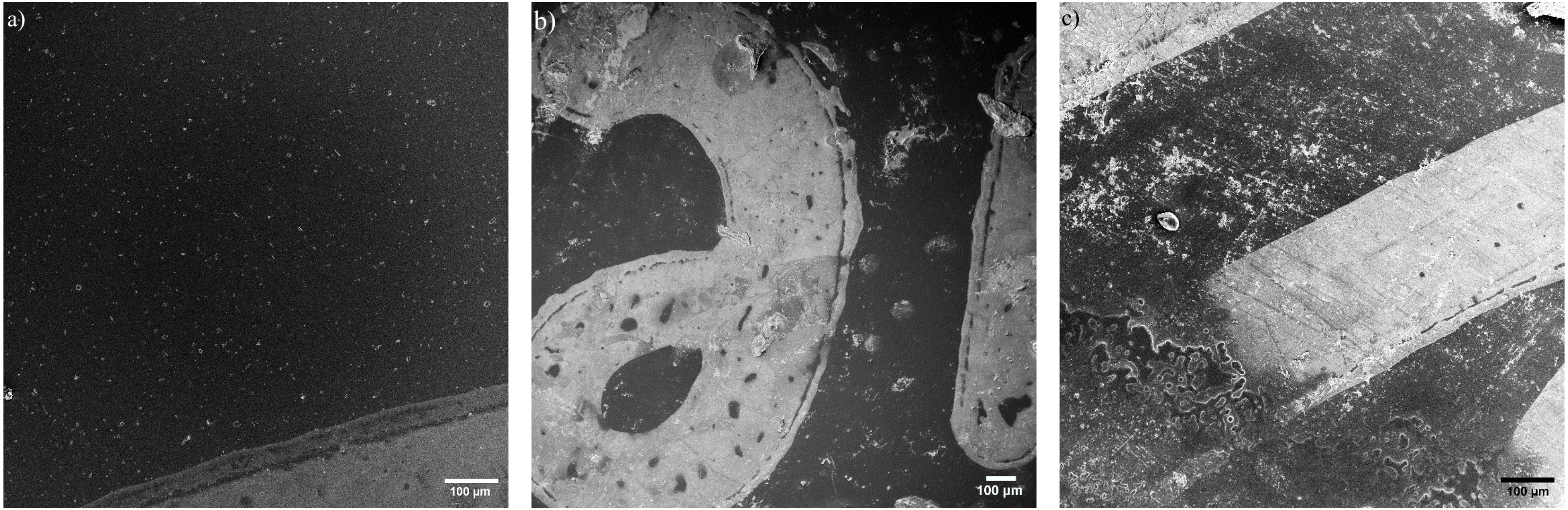

Supplementary Figure S5: Overview image of a a) original; b) 11-day composted and c) 25-day composted sticker. Signs of microbial colonisation and accumulation of organic residues can be seen on the two composted stickers (b & c). Possible mucilage formation is visible in the lower left part of the image (c).

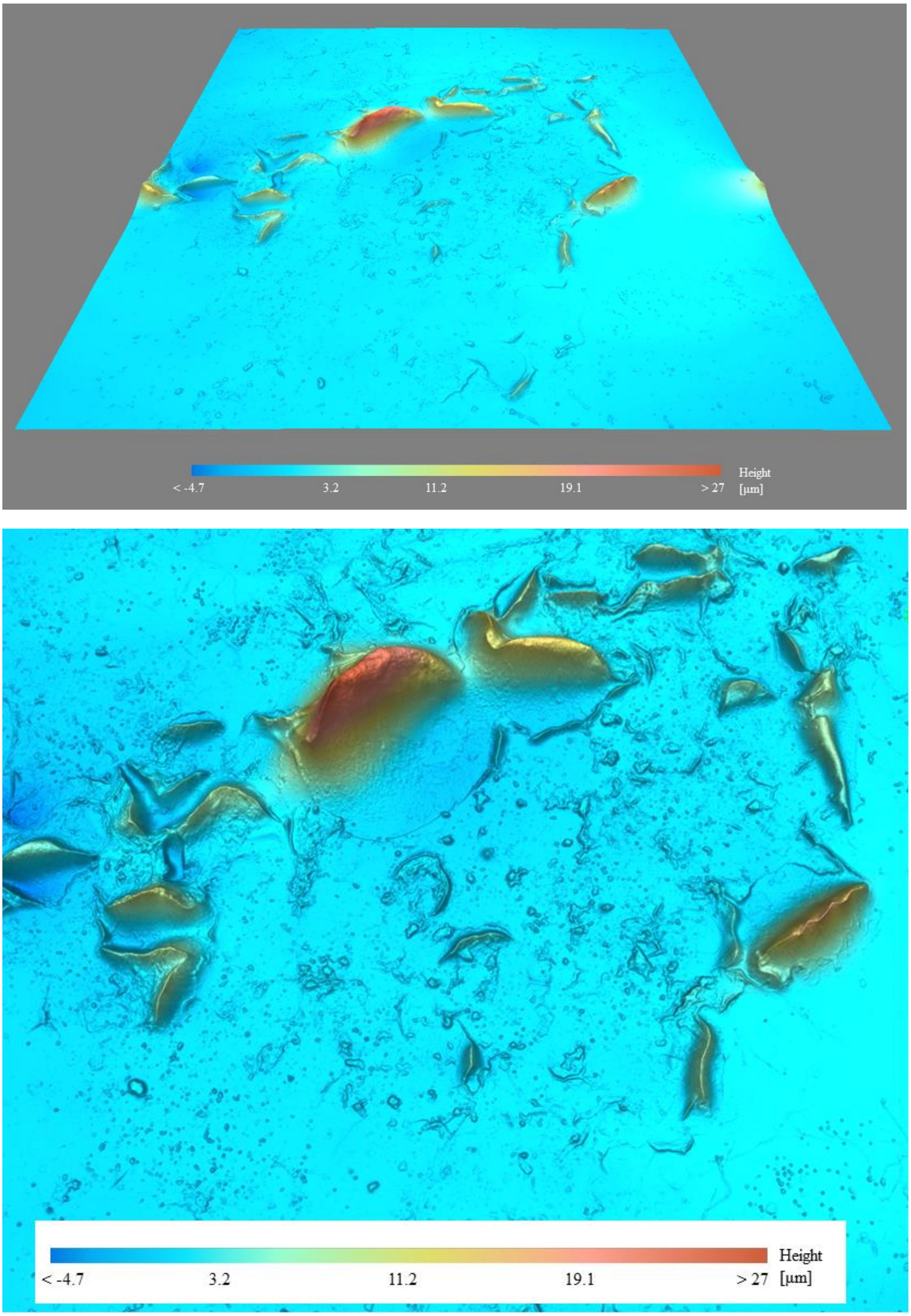

Supplementary Figure S6: Height of the delaminated area on the 25-day composted sticker based on the ESEM images using a 4-quadrant Si backscatter electron detector and analysis software (point electronic GmbH, Halle, Germany).

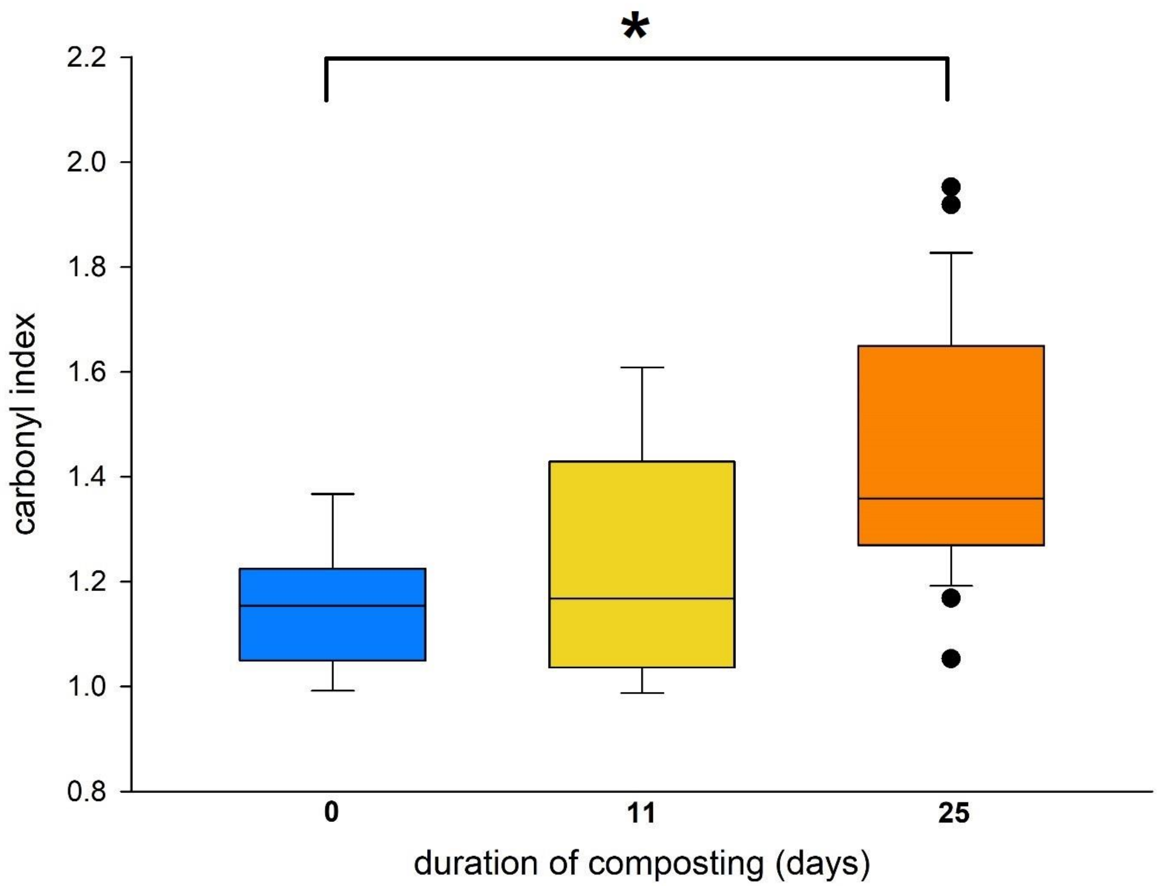

Supplementary Figure S7: Boxplots of the carbonyl indices of the original and composted stickers. Asterisk shows significant differences ($p < 0.05$) between non-composted and 25-day composted stickers (Kruskal-Wallis test followed by Dunn's test).

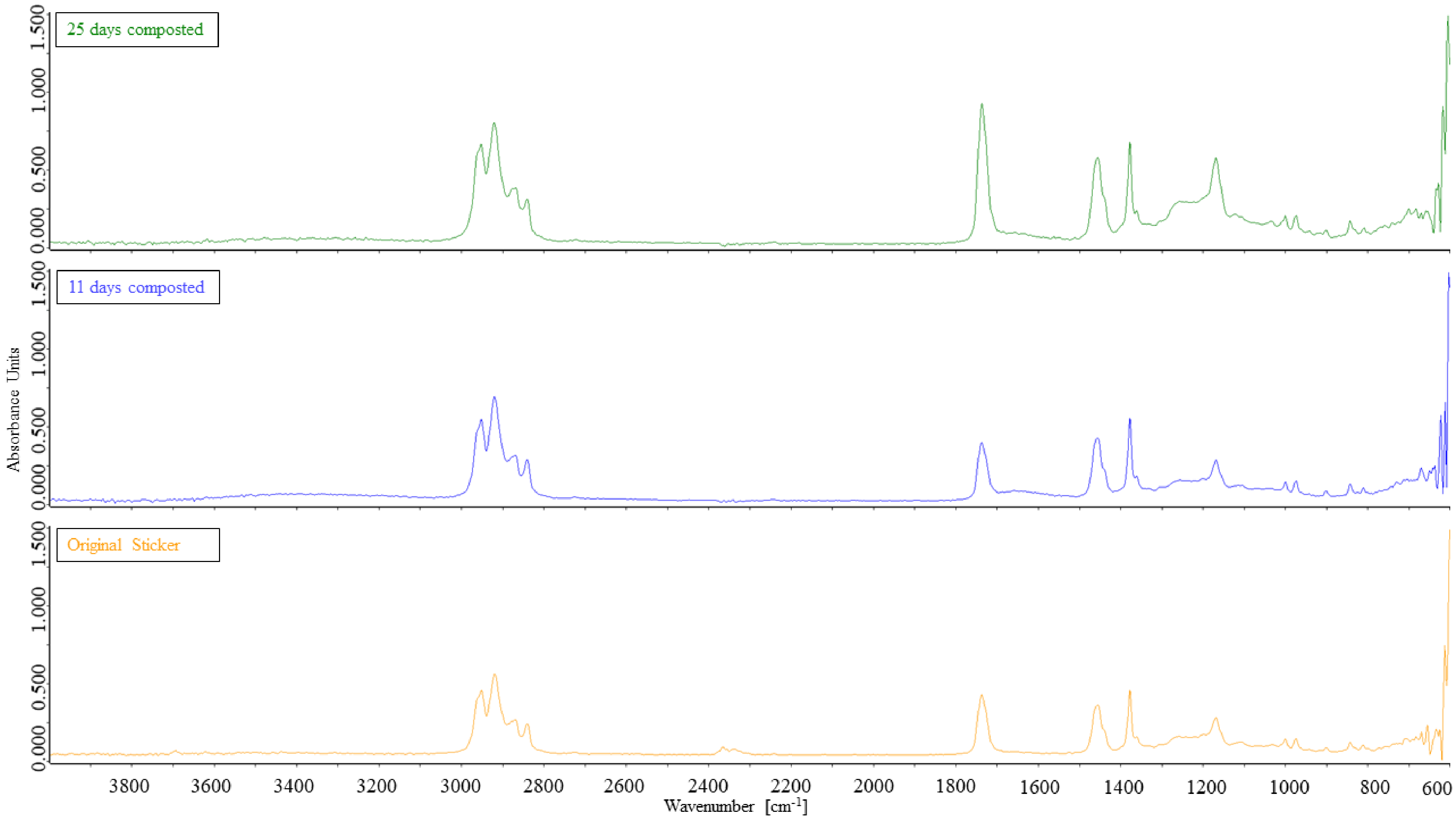

Supplementary Figure S8: FTIR-spectra of an original (bottom), 11 days composted (middle) and 25 days composted (top) sticker.

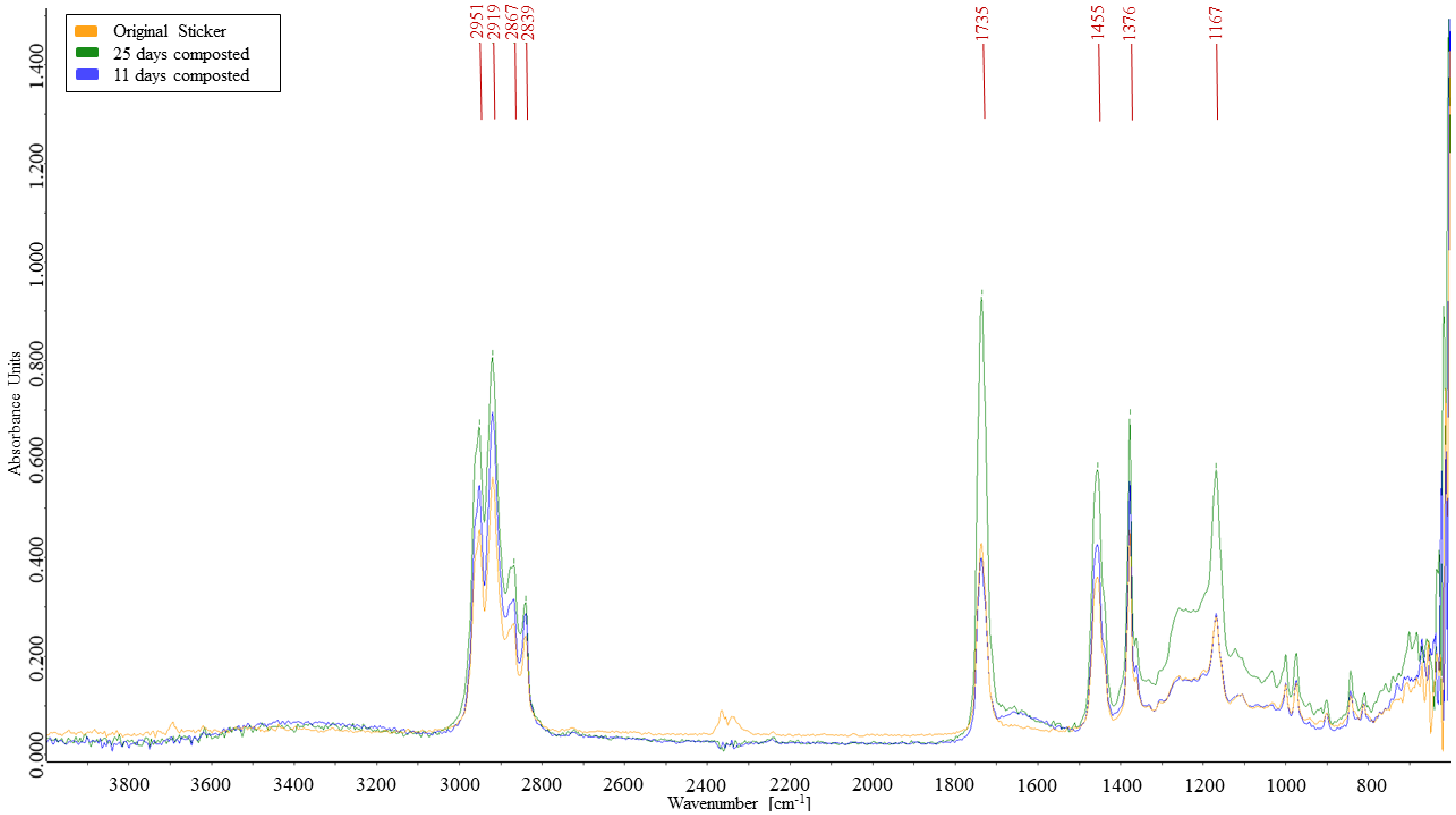

Supplementary Figure S9: FTIR-spectra of an original (orange), 11 days composted (blue) and 25 days composted (green) sticker and the corresponding peaks.

Supplementary Table S1: Experimental parameters for the micro- and nano-CT experiments and reconstruction datasets.

	**Micro-CT Original sticker**	**Micro-CT 25 days composted**	**Nano-CT Original sticker**	**Nano-CT 11 days composted**	**Nano-CT 25 days composted**
Sticker Preparation	scissors	scissors	scalpel	scalpel	laser
Current	80	80	25	25	25
Data Type	ushort	ushort	ushort	ushort	ushort
Dto R A Distance	13019.62109	13030.36328	1	1	1
Exp Time (s)	3	15	50	50	20
Field of view (µm)	371	371	65	65	65
Optical magnification	39.917	39.917	20	20	20
Pixel Size (µm)	0.724906	0.362358	0.063415	0.063415	0.127202
Voltage	50	50	35	35	35
Number of projections	2001	2001	901	901	501

Supplementary Table S2: Results of the connected component analysis of the micro-CT datasets in ORS Dragonfly.

**Micro-CT dataset**	**Region of interest**	**Voxel count**	**Volume (μm³)**	**Surface Area (μm²)**
Original sticker	Background	77439815	29499142.38	1161463.99
	Sticker	18049050	6875423.14	320545.04
	Attachment	16398	6246.49	4834.89
	Release paper	12997153	4951004.43	303111.83
25-day composted sticker	Background	620678131	29531125.93	1362107.65
	Sticker	153667275	7311305.85	349926.94
	Attachment	73983694	3520056.01	649451.31

Supplementary Table S3: Results of the connected component analysis of the nano-CT datasets in ORS Dragonfly.

Nano-CT dataset	Region of interest	Voxel count	Volume (µm³)	Surface Area (µm²)	Ratio of Porevolume : V. Pores/(V. Pores+ V. upper Sticker + V. lower Sticker)	Ratio of Attachmentvolume : V. Attach./(V. Attch.+ V. Pores+ V. upper Sticker + V. lower Sticker)
Original sticker	Upper Sticker	17658778	4440.19	5175.43	0.16	0
	Lower Sticker	68941306	17334.87	35335.11		
	Pores	16397525	4123.06	29896.52		
	Background	341537716	85877.59	17969.87		
	Attachment	0	0	0		
11-day composted Sticker	Upper Sticker	30343402	7738.21	5622.96	0.21	0.043
	Lower Sticker	111233565	28366.90	72520.06		
	Pores	38251065	9754.83	67689.40		
	Background	864588836	220488.33	30183.88		
	Attachment	1087132	277.24	2005.44		
25-day composted Sticker	Upper Sticker	8860892	13848.40	8949.57	0.26	0.042
	Lower Sticker	45324918	66206.89	206952.83		
	Pores	18932608	28576.81	200571.09		
	Background	52705506	70278.27	20082.06		
	Attachment	379980	582.81	4611.61		

Supplementary Table S4: Values for the calculation of the carbonyl index (CI) according to Almond et al. [33] based on the FTIR-spectra.

**Duration (days)**	**Container**	**Sticker**	**Area under band 1850-1650 cm^{-1}**	**Area under band 1500-1420 cm^{-1}**	**Carbonyl index=** $\frac{\text{Area under band } 1850-1650\ \text{cm}^{-1}}{\text{Area under band } 1500-1420\ \text{cm}^{-1}}$
0	Original Stickers	1	2.588	2.214	1.169
		2	2.921	2.137	1.367
		3	2.153	1.907	1.129
		4	2.369	2.081	1.138
		5	2.060	2.014	1.023
		6	2.445	2.069	1.182
		7	1.937	1.953	0.992
		8	2.614	2.110	1.239
11	1	1	2.026	2.052	0.987
	3	1	2.406	2.235	1.077
		2	2.159	2.083	1.036
	4	1	4.030	2.505	1.609
		2	3.303	2.312	1.429
	5	1	3.394	2.379	1.427
		2	2.397	2.053	1.168
25	2	1	2.997	2.204	1.360
		2	2.778	2.225	1.249
		3	3.846	2.935	1.310
	6	1	2.906	2.302	1.262
		2	2.739	2.223	1.232
		3	2.673	2.288	1.168
	7	1	2.925	2.269	1.289
		2	3.413	2.372	1.439
		3	4.383	2.440	1.796
		4	3.859	2.390	1.615
	8	1	3.186	2.363	1.348
		2	3.309	2.434	1.359
		3	3.144	2.355	1.335
		4	9.219	5.075	1.817
	9	1	3.183	2.345	1.357
		2	3.678	2.446	1.504
		3	4.075	2.825	1.442
		4	2.374	2.255	1.053
	10	1	5.761	2.951	1.952
		2	3.672	2.323	1.581
		3	4.899	2.927	1.674
		4	2.509	2.101	1.194
	11	1	3.061	2.333	1.312
		2	4.376	2.281	1.918
	12	1	2.740	2.193	1.249
		2	3.385	2.038	1.661
		3	2.637	1.983	1.330
		4	3.694	2.103	1.757

Supplementary Equation S1: Calculation of the carbonyl index according to Almond et al. (2020):

$$\text{Carbonyl index} = \frac{Area\ under\ band\ 1850{-}1650\ cm^{-1}}{Area\ under\ band\ 1500{-}1420\ cm^{-1}}\ .$$

Supplementary Equation S2: Calculation of the percentage of pore volume in the total sticker volume:

$$\text{Percentage of pore volume} = \frac{Volume\ pores}{(Volume\ pores + Volume\ upper\ sticker + Volume\ lower\ sticker)}$$
